# Supplementary material for: The Nursing Stress Scale-Spanish Version: An Update to Its Psychometric Properties and Validation of a Short-form Version in Acute Care Hospital Settings
Source: Int J Environ Res Public Health. 2020 Nov 15;17(22):8456. doi: 10.3390/ijerph17228456 (PMC7697776; doi:10.3390/ijerph17228456)
Supplement: Supplementary file 1 [file ijerph-17-08456-s001.pdf]

Supplementary Material

Table 1. Reporting Guideline Checklist. COSMIN checklist.

Items	Nursing Stress Scale – Spanish Version
General recommendations for the design of a study on measurement properties	+++
Content validity	NR
Structural validity	++
Internal consistency	+++
Cross-cultural validity/measurement invariance	NR
Measurement error and reliability	+++
Criterion validity	+++
Hypotheses testing for construct validity<br>A. Comparison with other outcome measurement instruments (convergent validity)	+++
B. Comparison between subgroups (discriminative or known-groups validity)	+++
Responsiveness	+++
A. Criterion approach (i.e. comparison to a 'gold standard')	+++
B. Construct approach (i.e. hypotheses testing; comparison with other outcome measurement instruments)	+++
C. Construct approach: (i.e. hypotheses testing: comparison between subgroups)	
D. Construct approach: (i.e. hypotheses testing: before and after intervention)	NR
Translation process	NR
